# The Radiological Spectrum of Rhino-Oculo-Cerebral Mucormycosis

**DOI:** 10.7759/cureus.40932

**Published:** 2023-06-25

**Authors:** Dinesh, Umakant Prasad, Sanjay K Suman, Manisha Kumari, Vaibhav Waghmare

**Affiliations:** 1 Radiodiagnosis, Indira Gandhi Institute of Medical Sciences (IGIMS), Patna, IND; 2 Radiology, Indira Gandhi Institute of Medical Sciences (IGIMS), Patna, IND

**Keywords:** rhino-oculo-cerebral mucormycosis, paranasal sinus, radiological spectrum, mucormycosis infection, fungal, rocm

## Abstract

Aim

We aim to study the spectrum of imaging findings in patients with rhino-oculo-cerebral mucormycosis (ROCM).

Materials and methods

This retrospective descriptive study was performed in histopathologically confirmed cases of rhino-oculo-cerebral mucormycosis in a tertiary care center in Bihar, India. The case records of patients with radiological, cultural, and histological evidence of acute invasive ROCM were retrospectively evaluated for relevant radiological and clinical data between May 2021 and June 2022.

Results

The radiological evaluation included computed tomography (CT) and magnetic resonance imaging (MRI) scans done on 52 patients. The patient's average age was 48 years. The ethmoid sinus was involved in 51 (98%) cases and the maxillary sinus in 50 (96%) cases. Bilateral sinus involvement (45, 86%) was the most common, followed by pansinus involvement (27, 52%). The orbit was involved in 39 (75%) cases, the face in 25 (47%) cases, and retroantral fat stranding in 24 (46%) cases. Mucosal thickening (91%) was the most common pattern of involvement, followed by complete opacification (77%). Osseous involvement was seen in 17 of 44 patients who had CT scans, and the majority of patients had extrasinus extension with intact bone. MRI revealed variable T2SI, with T2 hyperintensity being the most common pattern. Heterogeneous enhancement in post-contrast imaging was the most common.

Conclusion

ROCM is a life-threatening invasive fungal infection, especially in an immunocompromised state. ROCM is characterized by a variety of imaging abnormalities on CT and MRI, although nonspecific. Imaging aids in suspicion or early diagnosis in appropriate clinical contexts, particularly in an immunocompromised state, and in determining the degree of involvement and complications. Early detection of ROCM and its complications enables proper treatment, which can lower the cost of care, morbidity, and mortality.

## Introduction

Over more than 400,000 fungal species are known, and approximately 400 are human pathogens, only 50 of which cause systemic or central nervous system (CNS) infection. Most of these fungi are ubiquitous in our environment. Many people are colonized by fungi, but the infection is prevented by an intact immune system [1].

Rhino-oculo-cerebral mucormycosis (ROCM) is a life-threatening infection caused by saprophytic fungi belonging to the genera *Mucor*,* Rhizopus*,and* Absidia*. All of these belong to the order Mucorales and class Zygomycetes [2].

Rhinocerebral mucormycosis is seen almost exclusively in immunocompromised patients [2]. In the early stages, the disease typically presents with fever, headache, facial pain, nasal discharge, nasal obstruction, and crusting. The disease progresses rapidly within a period of a few hours to days leading to cranial nerve palsies and features of CNS involvement [2].

The disease is often misdiagnosed as a disease process (bacterial infection) involving the paranasal sinuses. It is a serious condition and is associated with a high mortality rate [1].

Early imaging is helpful in assessing the extent of involvement of this lethal disease, which requires prompt and aggressive treatment [2].

A huge surge in the number of coronavirus disease 2019 (COVID-19)-associated mucormycosis has been observed recently in India [3]. Imaging forms the cornerstone of management in patients with ROCM [3].

In patients with clinical suspicion and imaging evidence of ROCM, empirical antifungal therapy can be started even before the confirmation of the diagnosis by microbiology or histopathology [4].

In patients where a biopsy is planned, imaging can be used to help guide the site for biopsy to ensure maximum diagnostic yield. In patients with proven ROCM, imaging plays an important role in determining the extent of disease, which is critical in making a decision about the further line of management [3].

This article was previously posted to the medRxiv preprint server on May 2, 2023.

## Materials and methods

We retrospectively evaluated the imaging and clinical data of 52 patients (38 males and 14 females) who presented in the tertiary care hospital Indira Gandhi Institute of Medical Sciences (IGIMS), Patna, Bihar, India, aged 19-73 years old, with invasive mucormycosis of the rhino-oculo-cerebral areas. Patients were chosen for the study if they had a confirmed diagnosis of mucormycosis by biopsy and/or culture and if computed tomography (CT) scans or magnetic resonance imaging (MRI) were available for review over a period of two months during the second wave of COVID-19 between May 2021 and June 2022. Confirmed cases of mucormycosis by biopsy and/or culture but did not undergo imaging (CT or MRI) in our institute or suspected cases of ROCM on imaging but not confirmed by biopsy or culture were excluded.

The CT and/or MRI were retrieved from the picture archiving and communication system (PACS).

CT scan was performed on Aquilion 64 TSX-101A (Toshiba Medical Systems, Tokyo, Japan) or Aquilion Lightning, Canon Medical Systems, 16-row 32 slice (Canon Medical Systems, Otawara, Tochigi, Japan) machine using a routine CT paranasal sinus (PNS) and/or skull protocol with 130 kVP and 150-220 mA tube current. Intravenous contrast medium (low osmolar, non-ionic, 350 mg/mL iodine content) was used routinely at a dose of 1 mL/kg administered by a pressure injector. Conventional MR PNS and/or brain and/or orbit imaging including axial, coronal, and sagittal T1-weighted, T2-weighted, and fat-suppressed T1-weighted images after intravenous injection of gadopentetate dimeglumine (0.1 mmol/kg) were acquired. MRI was performed using GE OPTIMA MR360 1.5T (General Electric Healthcare, Milwaukee, WI, USA).

Image analysis

Three staff radiologists conducted a consensus retrospective review to look for sites and extent of involvement, signal characteristics, and complications.

Image interpretation

Sinus involvement in CT or MRI was recorded in each case. Three patterns of sinus involvement on CT were recorded: mucosal thickening, complete opacification, and air-fluid level. The involvement of bone was evaluated on CT. Signal intensities on T1-weighted and T2-weighted images were recorded. Fat stranding and soft tissue extension that resembled the intrasinus soft tissue were interpreted as indicators of involvement in the orbit, retroantral, masticator, and pterygopalatine.

Cavernous sinus and internal carotid artery (ICA) involvement were seen as thickening and non-enhancement on post-contrast scans and loss of flow void in ICA in MRI. Dural enhancement, the presence of extradural collections, infarcts, cerebritis, and intracerebral abscess were all evaluated in patients with intracranial extension.

Statistical analysis

Categorical data has been presented in percentages in statistical analysis.

## Results

A total of 52 patients were selected for the radiological evaluation, of whom 44 underwent CT, 25 underwent MRI, and 17 underwent both. Non-contrast computed tomography (NCCT) was performed in 34 cases, contrast-enhanced computed tomography (CECT) in 10, and plain MRI in 11, while 14 underwent cardiac magnetic resonance imaging (CMRI).

The picture archiving and communication system (PACS) was used to retrieve the images from computed tomography (CT) and/or magnetic resonance imaging (MRI).

Demographic and clinical background

Our study group included 38 males and 14 females ranging in age from 19 to 75 years (mean: 48 years). Patients over the age of 40 made up the majority (78.8%), with people in their 40s and 60s (65%) suffering the most.

Out of 52 patients selected, 47 (90.4%) patients were diabetic, 32 (61.5%) patients had a recent (within the last two months) history of COVID-19, 24 (46.2%) patients had recent systemic (oral prednisolone or IV methylprednisolone) steroid use for a median duration of 14 days, and 17 (32.7%) patients had a history of nasal oxygen use. Out of 52 cases, 46 were histopathologically proven, and the remaining six were microbiologically proven (culture positive) (four were *Mucor* and two were *Rhizopus*).

Imaging findings

Sinonasal Involvement

The ethmoid sinus was the most common sinus involved in our study (51, 98%), followed by the maxillary sinus (50, 96%), and the frontal was the least commonly involved (40, 77%). Bilateral sinus involvement (45, 86%) was a more common finding as compared to unilateral involvement. With the exception of one case with isolated maxillary involvement, the majority of patients (27, 52%) had pansinusitis, followed by unilateral involvement of the ethmoid, maxillary, sphenoid, and frontal sinuses (11, 21%), and the remaining cases had multiple sinus involvement. Details of sinus involvement are shown in Table 1.

**Table 1:** Paranasal sinus involvement

Sinus involved	Number (%)
Maxillary	50 (96%)
Ethmoid	51 (98%)
Sphenoid	41 (79%)
Frontal	40 (77%)
Pansinusitis	27 (52%)

Extrasinus Involvement

**Figure 1:**
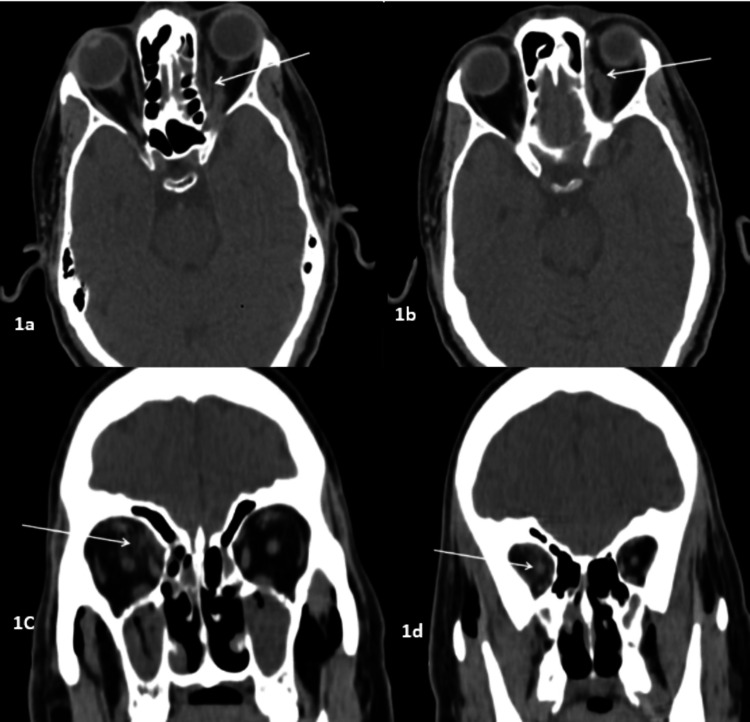
Orbital involvement in different patients 1a and 1b: Axial CT image of a 57-year-old diabetic patient with ROCM showing left intra- and extraconal fat stranding with soft tissue opacification of the left orbital apex (arrow) and thickening of the left superior ophthalmic vein (arrow). 1c and 1d: Coronal CT scan of another 68-year-old patient with ROCM showing right intra- and extraconal fat stranding (arrow) with thickening of the right optic nerve (arrow). CT: computed tomography, ROCM: rhino-oculo-cerebral mucormycosis

**Figure 2:**
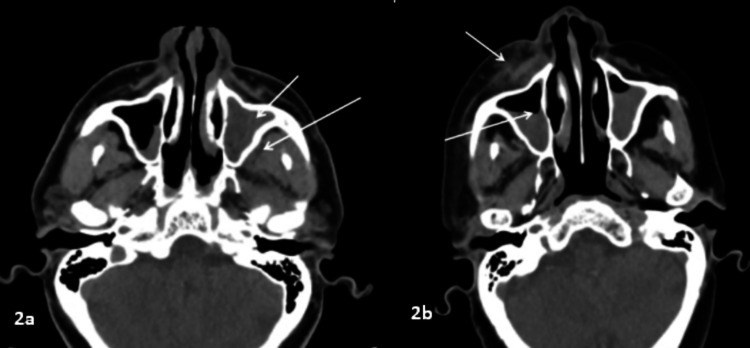
Periantral involvement in different patients 2a: Axial CT scan of a 52-year-old patient with mucormycosis showing complete sinus opacification of the left maxillary sinus (short arrow) with retroantral fat stranding (long arrow). 2b: Axial CT scan of a 57-year-old patient with mucormycosis showing air-fluid level in the right maxillary sinus (long arrow) with preantral fat standing (short arrow). Both images show periantral involvement with an intact bony wall, suggestive of the perineural spread of the infection. CT: computed tomography

The orbit was the most common site involved in our study, in which extraconal fat stranding or soft tissue opacification and/or altered signal intensity were most commonly seen (39, 75%) (Figure 1), followed by extraocular muscle thickening or altered signal intensity (22, 42%). Face involvement was seen in 25 (47%) patients, and retroantral fat stranding was seen in 24 (46%) patients (Figure 2). Optic nerve thickening or altered signal intensity was observed in 10 (19%) patients (Figure 1d). Other extrasinus involved sites were the pterygopalatine, infratemporal fossa, and optic nerve. Intracranial involvement included the cavernous sinus, meninges, brain parenchyma, and internal carotid artery. Tables 2-4 provide additional information on extrasinus extension, including the type of involvement.

**Table 2:** Orbital structure involvement SI: signal intensity

Orbital structures involved	Number (%)	Pattern
Extraconal compartment	39 (75%)	Fat stranding or abnormal SI
Intraconal compartment	19 (36%)	Fat stranding or abnormal SI
Extraocular muscle involvement	22 (42%)	Thickening or abnormal SI or enhancement
Optic nerve	10 (19%)	Thickening or abnormal SI or enhancement
Globe tenting (guitar pick sign)	9 (17%)	
Exophthalmos	6 (11.5%)	

**Table 3:** Extrasinus involvement other than orbital and intracranial structure

Structures involved	Number (%)
Face/preantral	25 (48%)
Retroantral	24 (46%)
Infratemporal fossa	16 (31%)
Pterygopalatine fossa	20 (38%)
Skull bone	3 (0.6%)

**Table 4:** Intracranial involvement SI: signal intensity

Structures involved	Number (%)	Pattern
Cavernous sinus	3 (0.6%)	Filling defect or abnormal SI
Meninges	2 (0.4%)	Meningeal enhancement
Brain parenchyma	2 (0.4%)	Infarction and cerebritis
Internal carotid artery	2 (0.4%)	Loss of flow void and vessel luminal attenuation

Imaging Appearance and Signal Characteristics

**Figure 3:**
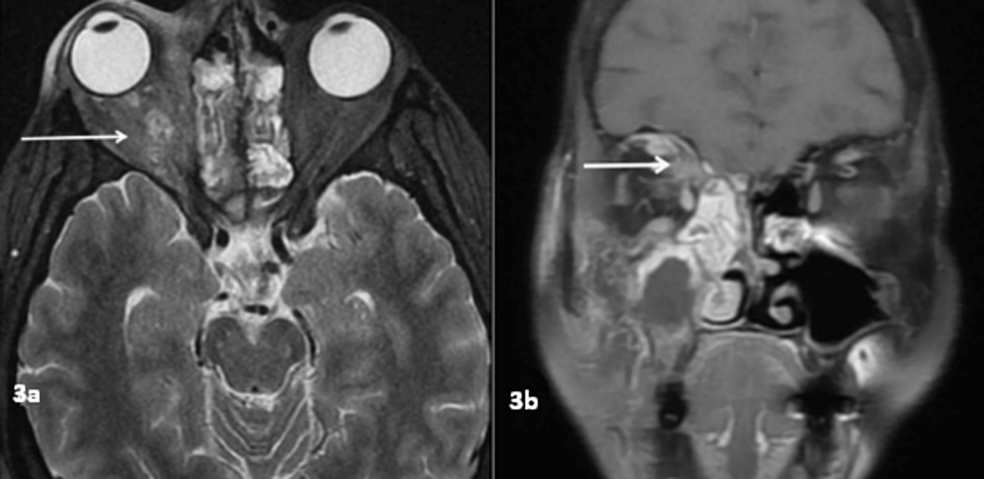
Orbital involvement 3a: Coronal T2WI of a 43-year-old patient with ROCM showing altered signal intensity in the right intra- and extraconal compartment (arrow). 3b: Coronal post-contrast FS T1WI showing abnormal enhancement of the right intra- and extraconal fat (arrow). T2WI: T2-weighted image, ROCM: rhino-oculo-cerebral mucormycosis, FS T1WI: fat-suppressed T1-weighted image

**Figure 4:**
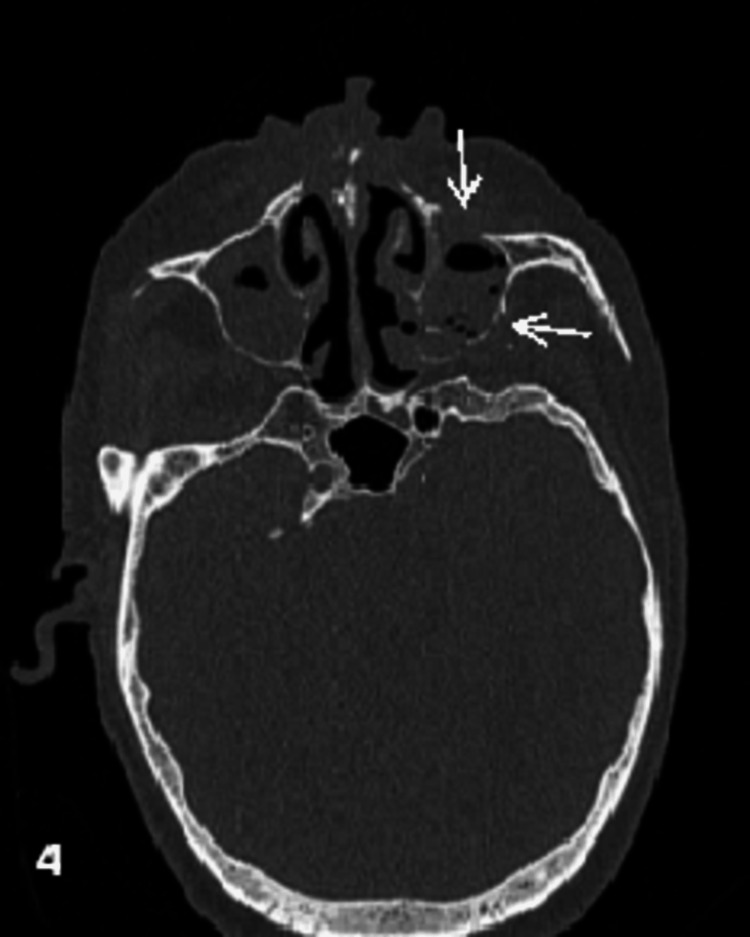
Osseous involvement Axial CT bone window image of a 65-year-old diabetic patient who presented with facial pain and redness showing erosion of the anterior and posterior wall of the left maxillary sinus (arrows). CT: computed tomography

**Figure 5:**
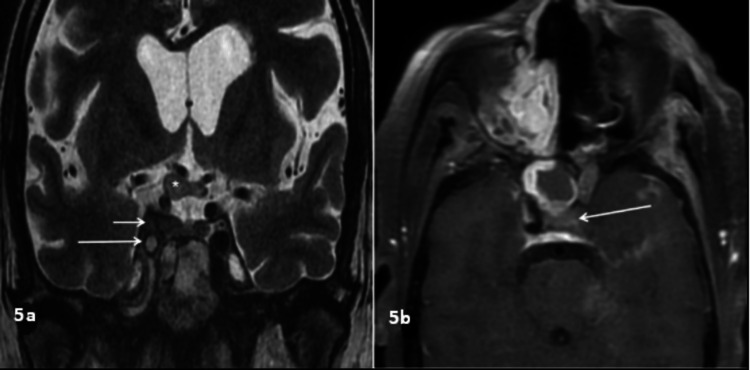
Cavernous sinus involvement 5a: Coronal T2WI of a 42-year-old patient who had recent COVID-19 and systemic steroid use who presented with headache and right eye vision loss showing loss of flow of void in the right ICA (large arrow) with abnormal signal intensity in the right cavernous sinus surrounding the right ICA (small arrow) and thickening of the right optic nerve and optic chiasma (asterisk). 5b: Axial post-contrast FS T1WI of a 50-year-old patient who had recent COVID-19 and systemic steroid use who presented with headache and left eye vision loss showing loss of flow void in the left ICA (arrow). T2WI: T2-weighted image, COVID-19: coronavirus disease 2019, ICA: internal carotid artery, FS T1WI: fat-suppressed T1-weighted image

**Figure 6:**
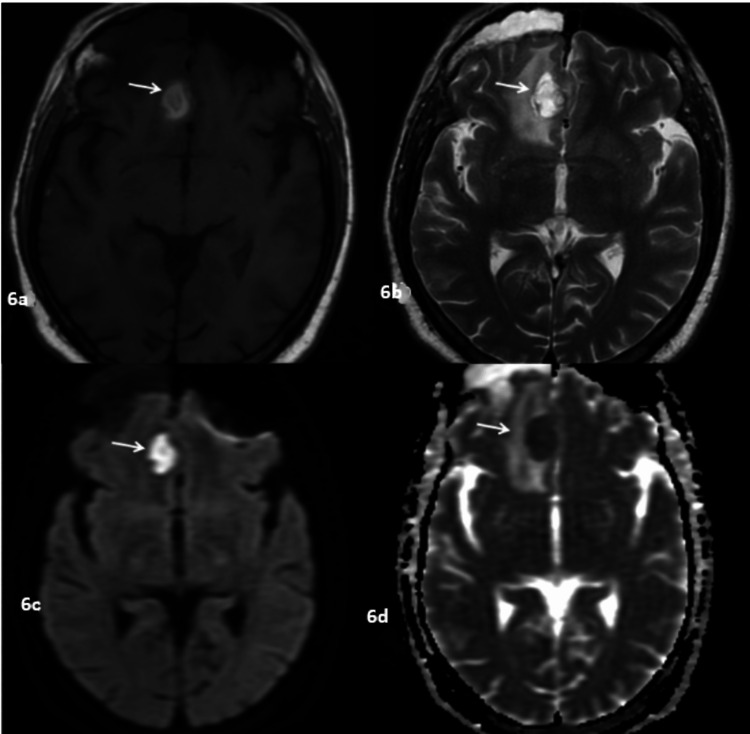
Brain abscess in a 68-year-old diabetic patient with a history of recent systemic steroid use who presented with nasal symptoms and headache 6a: Axial T1WI showing hyperintense lesion in the right gyrus rectus (arrow). 6b: Axial T2WI showing that the lesion is hyperintense on T2WI as well with adjacent edematous changes (arrow). 6c: Axial DWI showing that the lesion shows diffusion restriction (arrow). 6d: ADC map showing that the lesion is hypointense, suggestive of true restriction (arrow). T1WI: T1-weighted image, T2WI: T2-weighted image, DWI: diffusion-weighted imaging, ADC: apparent diffusion coefficient

**Figure 7:**
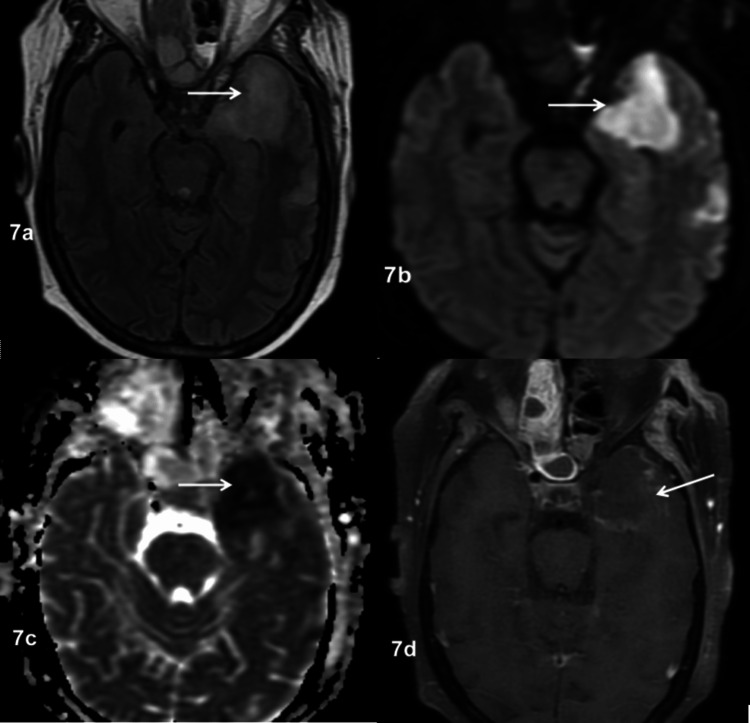
Subacute infarct in a patient with ROCM 7a: Axial FLAIR image showing hyperintensity in the left frontal lobe (arrow). 7b: Axial DWI at b-1000 showing diffusion restriction in the left frontal lobe (arrow). 7c: ADC map showing hypointensity in the corresponding region (arrow). 7d: Axial post-contrast FS T1WI showing mild peripheral enhancement, suggestive of a subacute infarct (arrow). ROCM: rhino-oculo-cerebral mucormycosis, FLAIR: fluid-attenuated inversion recovery, DWI: diffusion-weighted imaging, ADC: apparent diffusion coefficient, FS T1WI: fat-suppressed T1-weighted image

Mucosal thickening (91%) was the most common pattern of sinus involvement, followed by complete sinus opacification (77%), and air-fluid level (34%) (Figure 2). Hyperdense content within the sinuses was noted in seven patients. A combination of patterns was most commonly seen in individual patients. Nasal cavity involvement (46, 88%) was seen as nonspecific mucosal thickening, fluid or soft tissue content in the nasal cavity, and heterogeneous or non-enhancing nasal mucosa. Intraconal or extraconal orbital involvement was observed in the form of fat stranding, soft tissue opacification, or altered signal intensity (Figures 1, 3). Extraocular muscle and optic nerve involvement were found to have thickened or altered signal intensity. Exophthalmos and globe tenting were observed as a result of increased orbital tension caused by optic nerve stretching of the posterior globe. Extrasinus involvement was observed in 39 patients out of 44 who underwent CT, whereas sinus wall involvement was seen in only 16 patients, suggesting extrasinus spread of disease through intact bone. Bone involvement in the form of bone rarefaction, erosion, and permeative destruction was seen in 16 patients, involving the sinus wall (16) and skull base (3) (Figure 4). Face, retroantral, infratemporal fossa, and pterygopalatine fossa involvement were observed in the form of fat stranding, altered signal intensity, or abnormal enhancement. Cavernous sinus filling defect or abnormal enhancement was observed in cavernous sinus involvement in two patients (Figure 5). Abnormally enhancing meninges were observed in two patients. Loss of flow void and filling defect were seen in the internal carotid artery. Brain parenchymal involvement was seen as brain abscess, infarction, and cerebritis (Figures 6, 7).

**Figure 8:**
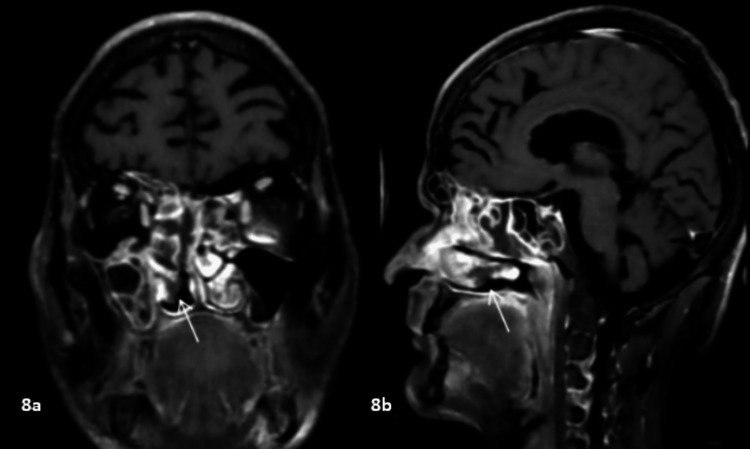
Black turbinate sign 8a and 8b: Coronal and sagittal post-contrast FS T1WI showing non-enhancing right inferior turbinate (arrows). FS T1WI: fat-suppressed T1-weighted image

Homogeneous enhancement of the mucosa was observed in four patients, heterogeneous enhancement was seen in five patients, and one patient showed no enhancement in a total of 14 patients who underwent CECT. Six patients showed homogeneous enhancement, and eight patients had heterogeneous enhancement with non-enhancing areas on post-contrast fat-suppressed T1-weighted images in 14 patients who underwent contrast-enhanced MRI. The black turbinate sign refers to the non-enhancement of nasal turbinates in a patient with acute invasive fungal rhinosinusitis, which was observed in five patients in our study (Figure 8). Details of patterns of disease, signal intensity, and enhancement patterns are enlisted in Tables 5-7.

**Table 5:** CT features (34 NCCT and 10 CECT) CT: computed tomography, NCCT: non-contrast computed tomography, CECT: contrast-enhanced computed tomography

Pattern on CT	Number (%)
Mucosal thickening	40 (91%)
Air-fluid level in sinuses	15 (34%)
Sinus opacification	34 (77%)
Hyperdense content in sinuses	7 (16%)
Osseous involvement	17 (38%)

**Table 6:** CECT enhancement pattern CECT: contrast-enhanced computed tomography, CT: computed tomography

Enhancement pattern in CT	Total (10 patients)
Non-enhancing	1 (10%)
Mild homogeneous enhancement	4 (40%)
Heterogeneous enhancement	5 (5%)

**Table 7:** MRI features (11 plain and 14 contrast enhanced) MRI: magnetic resonance imaging

T1 weighted	T2 weighted	Enhancement pattern (number 14)
Hypointense (25 (100%))	Hyperintense (15 (60%))	Homogeneous enhancement (6 (43%))
Hyperintense content (5 (20%))	Isointense (6 (24%))	Heterogeneous enhancement (8 (57%))
	Hypointense (4 (16%))	Black turbinate sign (non-enhancement) (5)
	Hypointense content in the sinus (9 (36%))	

## Discussion

Mucormycosis is an invasive fungal infection that was first described in 1885 by Paulltauf [4]. Although it can affect various body organs, the rhinocerebral form is the most common [5]. These organisms can become pathogenic in immunocompromised patients, as well as those with poorly controlled diabetes mellitus and diabetic ketoacidosis. Organ transplantation, hematologic malignancies, chronic corticosteroid treatment, and hemochromatosis are all examples of immunocompromised states [5]. Forty-seven patients in our study were diabetic, 32 patients had recent COVID-19, and the majority of them received systemic steroids, making them susceptible to mucormycosis.

The infection begins in the nasal cavity and spreads to the paranasal sinuses. Early fungal implantation is typical in the maxillary sinus, where there is no bone degradation. The middle turbinate is the most commonly involved site in mucor, followed by the middle meatus and septum [6].

Fungi can spread through perivascular channels beyond paranasal sinuses with intact bony walls (Figure 1) [7].

It virtually penetrates all of the tissues nearby. There is a fulminant progression over a few days to many weeks; it advances to the brain either through angioinvasion or ethmoid sinuses, orbital apex, and bone erosion [1,6]. Intracranial infection spread predicts increased mortality and morbidity, with up to 73% of patients dying [1]. Imaging aids in the assessment of osseous destruction, which is followed by intracranial, cavernous sinus, and intraorbital extension. However, early radiological changes, such as mucosal thickening, are indistinguishable from nonspecific sinusitis on either MRI or CT. A high index of suspicion and early diagnosis are crucial, especially in immunocompromised patients. [7].

Although sinus CT is the preferred imaging modality for evaluating signs of invasion, bone destruction is frequently observed late in the infection course after soft tissue necrosis. Magnetic resonance imaging is more sensitive in identifying the intradural and intracranial extent of ROCM, cavernous sinus thrombosis, and thrombosis of the internal carotid artery's cavernous portions [8].

Middlebrooks et al. [9] proposed a seven-variable CT-based model that was found to be useful as a screening tool to identify patients at risk for acute invasive fungal sinusitis. The pterygopalatine fossa, bone dehiscence, orbital invasion, septal ulceration, lacrimal sac, and periantral fat were among the factors. In their study, the involvement of periantral fat was the best individual predictor of invasive fungal sinusitis.

Patel et al. [10] found bony erosion in 75% of COVID-19-ROCM patients, whereas DelGaudio et al. [11], Gupta et al. [12], and Therakathu et al. [2] found bony erosion in only 33%-40% of pre-COVID-19-ROCM cases. Osseous involvement in our study was seen in 38% of cases in the form of bone erosions (most common), rarefaction, and permeative destruction. Erosions were frequently found in the walls of the maxillary antrum and orbital walls, allowing them to spread into the retromaxillary soft tissue and orbit, respectively. CT images helped visualize the erosion or rarefaction of bony walls.

In fungal sinusitis, hyperdense intraluminal contents represent fungal hyphae and debris [1]. According to Patel et al. [10], approximately one-third of patients with ROCM had hyperdense material, but we observed this finding in 16% of instances.

Due to the angio-invasiveness of fungi, extrasinus involvement can occur without bony erosions by spreading through small vascular channels [2,13]. This finding was seen in 20 (45%) patients in our study.

Another early sign of ROCM is superficial cellulitis, as the involvement of the superficially located subcutaneous fat is uncommon in non-fungal sinusitis. Patel et al. [10] found superficial cellulitis in 35% of cases, with premaxillary fat stranding/soft tissue replacement being the most common. In our study, 48% of patients had superficial cellulitis in our study.

Regarding sinuses, Agarwal et al. [14] observed variable signal intensity on T2WI and predominantly (90%) hypointensity on T1WI. In our study, we also found variable signal intensity on T2WI (60% hyperintense, 25% isointense, and 4% hypointense), and all showed hypointensity on T1WI. The accumulation of hemosiderin and paramagnetic materials such as calcium, iron, magnesium, and manganese has been linked to hypointensity on T1WI and T2WI in fungal disease [2,14,15].

ROCM is also characterized by periantral fat invasion, which can manifest as stranding of premaxillary/retroantral fat, increased signals on fat-saturated T2-weighted imaging, or enhancement on contrast sequences. Fat stranding can also be seen on CT images. In our study, 59.6% of patients had periantral fat involvement. Agrawal et al. [15] showed periantral involvement in 75.83% of cases.

In the early stages, there is intense homogeneous enhancement of the mucosa, similar to bacterial sinusitis. The presence of heterogeneous enhancement or non-enhancing areas within the lesion suggests that it is caused by an invasive fungal etiology. The disease is known to devitalize the sinonasal mucosa through vascular invasion and infarction, resulting in non-enhancing tissues on contrast administration [15].

Only five of the 14 patients with available post-contrast MRI had the black turbinate sign described by Safder et al. [16] as a feature of early nasal mucormycosis.

Limitations of the study

Single-centered and retrospective nature was the limitation of our study. All patients did not undergo a contrast-enhanced study, which could have better evaluated the disease process. Multicenter studies in the future may overcome the limitations faced in our study.

## Conclusions

ROCM is a life-threatening invasive fungal infection, especially in an immunocompromised state. ROCM is characterized by a variety of imaging abnormalities on CT and MRI, although nonspecific. Imaging aids in suspicion or early diagnosis in appropriate clinical contexts, particularly in an immunocompromised state, and in determining the degree of involvement and complications. Early detection of ROCM and its complications enables proper treatment, which can lower the cost of care, morbidity, and mortality.
